# Insights into Genes Encoding LEA_1 Domain-Containing Proteins in *Cyperus esculentus*, a Desiccation-Tolerant Tuber Plant

**DOI:** 10.3390/plants13202933

**Published:** 2024-10-19

**Authors:** Yongguo Zhao, Xiaowen Fu, Zhi Zou

**Affiliations:** 1 College of Biology and Food Engineering, Guangdong University of Petrochemical Technology, Maoming 525000, China; zhaoyongguo@gdupt.edu.cn; 2 National Key Laboratory for Tropical Crop Breeding, Hainan Key Laboratory for Biosafety Monitoring and Molecular Breeding in Off-Season Reproduction Regions, Institute of Tropical Biosciences and Biotechnology of Chinese Academy of Tropical Agricultural Sciences, Haikou 571101, China; xiaowen9924@126.com; 3 Sanya Research Institute of Chinese Academy of Tropical Agricultural Sciences, Sanya 572024, China

**Keywords:** tigernut, vegetative tissue, desiccation tolerance, late-embryogenesis-abundant protein, LEA_1 domain-containing protein, expression divergence

## Abstract

LEA_1 domain-containing proteins constitute a class of late-embryogenesis-abundant proteins that are highly hydrophilic and predominantly accumulate in mature seeds. Though LEA_1 proteins have been proven to be essential for seed desiccation tolerance and longevity, little information is available on their roles in non-seed storage organs. In this study, a first genome-wide characterization of the *LEA_1* gene family was conducted in tigernut (*Cyperus esculentus* L., Cyperaceae), whose underground tubers are desiccation tolerant with a moisture content of less than 6%. Five family members identified in tigernut are comparative to four to six found in seven other Cyperaceae plants, but relatively more than three reported in Arabidopsis. Further comparison of 125 members from 29 plant species supports early divergence of the *LEA_1* family into two phylogenetic groups before angiosperm radiation, and gene expansion in tigernut was contributed by whole-genome duplications occurring after the split with the eudicot clade. These two phylogenetic groups could be further divided into six orthogroups in the momocot clade, five of which are present in tigernut and the remaining one is Poaceae specific. Frequent structural variation and expression divergence of paralogs were also observed. Significantly, in contrast to seed-preferential expression of *LEA_1* genes in Arabidopsis, rice, and maize, transcriptional profiling and qRT-PCR analysis revealed that *CeLEA1* genes have evolved to predominantly express in tubers, exhibiting a seed desiccation-like accumulation during tuber development. Moreover, *CeLEA1* transcripts in tubers were shown to be considerably more than that of their orthologs in purple nutsedge, another Cyperaceae plant producing desiccation-sensitive tubers. These results imply species-specific activation and key roles of *CeLEA1* genes in the acquisition of desiccation tolerance of tigernut tubers as observed in orthodox seeds. Our findings not only improve the understanding of lineage-specific evolution of the *LEA_1* family, but also provide valuable information for further functional analysis and genetic improvement in tigernut.

## 1. Introduction

Desiccation tolerance is an ancient trait that appeared very early in the evolution of terrestrial life. Nevertheless, in vascular plants, this trait is confined to spores, pollen, and seeds, but has usually been lost from vegetative tissues [1]. To date, the mechanisms of desiccation tolerance have been extensively studied in seeds, which could be divided into three types on the basis of their sensitivity to desiccation, i.e., orthodox, intermediate, and recalcitrant [2]. Among them, orthodox seeds can survive ex situ storage for very long periods under conventional gene-bank conditions, and the underlying mechanisms are usually associated with significant accumulation of late-embryogenesis-abundant (LEA) proteins, heat-shock proteins (HSPs), antioxidant enzymes, and non-reducing oligosaccharides [1,3,4]. LEA proteins, which are usually small and extremely hydrophilic, were first discovered in *Gossypium hirsutum* seeds and then also in vegetative tissues especially under stress conditions [5,6,7]. Thus far, up to eight families with defined Pfam domains have been described, which includes the LEA_1 family under the accession number of PF03760 [8]. This family is also known as D-113 or Group 4 LEA, which features a conserved LEA_1 domain of 70 to 80 residues at the N-terminal [7,9,10]. This region is necessary and sufficient for folding into α-helix, which is required for chaperone-like activity under water limitation [11]. By contrast, the C-terminal region is highly diverse with variable size and random coil structure [6]. Genome-wide survey revealed that the model plant Arabidopsis (*Arabidopsis thaliana*) contains three *LEA_1* genes, i.e., *AtLEA6*, *AtLEA18*, and *AtLEA46* [7,12]. An interesting phenomenon is that all of them exhibit a seed-preferential expression pattern, though *AtLEA46* transcripts in leaves were shown to be significantly induced by various stresses such as cold, drought, salt, heat, and powdery mildew [7,13]. Overexpressing *AtLEA46* could confer tolerance to severe drought of transgenic Arabidopsis plants, whereas knockdown mutants in this family are sensitive to water deficit, showing a reduced number of floral and axillary buds as well as a reduced seed production under optimal irrigation relative to wild-type plants [13]. Involvement of this family in the acquisition of seed desiccation tolerance and longevity was supported by comparative proteomic analyses [14,15]. By contrast, roles of LEA_1 proteins in desiccation tolerance of non-seed storage organs such as underground tubers and tuberous roots have not been well studied.

Tubers and tuberous roots, whose development usually does not undergo dehydration, possess up to 70% water and are highly sensitive to desiccation [16]. A rare exception is yellow nutsedge or tigernut (*Cyperus esculentus* L.), whose seed tubers are desiccation tolerant with a moisture content of less than 6% [17,18,19]. Tigernut belongs to the Cyperaceae family within Poales and is a unique plant accumulating up to 35% of oil in underground tubers, which are developed from stolons [20,21,22,23]. The tuber development could be divided into three main stages, i.e., initiation, swelling, and maturation [17,19,22]. Whereas two early stages are characterized with a water content of approximately 85%, a sharp drop to less than 45% could be observed during maturation [17,19]. Interestingly, during tuber maturation, a seed desiccation-like accumulation of oleosins and seed maturation proteins (SMPs) was also observed [17,22], implying their putative roles in the acquisition of desiccation tolerance.

In this study, we present a first genome-wide characterization of *LEA_1* family genes in tigernut, including gene structures, sequence features, duplication events, evolution patterns as well as expression profiles. Significantly, our results support early divergence of the *LEA_1* family into two phylogenetic groups before angiosperm radiation, and *CeLEA1* genes have evolved to predominantly express in tubers, exhibiting a seed desiccation-like accumulation during tuber development. These findings will provide valuable information for further functional analyses.

## 2. Results

### 2.1. Characterization of Five LEA_1 Family Genes in Tigernut

**Figure 1:**
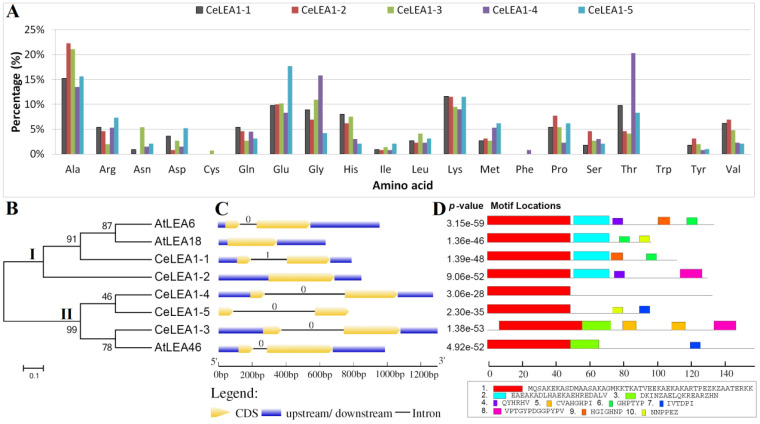
Structural and phylogenetic analyses of *LEA_1* family genes in *C. esculentus*. (**A**) Amino acid composition of CeLEA1 proteins. (**B**) The unrooted phylogenetic tree resulting from full-length Ce/AtLEA1 proteins with MEGA6 (maximum likelihood method and bootstrap of 1000 replicates), where the distance scale denotes the number of amino acid substitutions per site. The name of each clade is indicated next to the corresponding group. (**C**) Exon–intron structures, where 0 and 1 indicate intron phases. (**D**) The distribution of conserved motifs among Ce/AtLEA1 proteins, where different motifs are represented by different color blocks as indicated and the same color block in different proteins indicates a certain motif. (At: *A. thaliana*; CDS: coding sequence; Ce: *C. esculentus*; LEA: late embryogenesis abundant).

In a previous study, functional annotation of the full-length transcriptome of tigernut resulted in a high number of genes encoding LEA proteins, four of which belong to the LEA_1 family [24]. To provide a global view of the whole family, we took advantage of the recently available genome [25] to identify the complete set of *CeLEA1* genes. As shown in Table 1, five members, named *CeLEA1-1–5*, were identified from five scaffolds (Scfs), i.e., Scf2, Scf11, Scf21, Scf22, and Scf23. Interestingly, the family amounts are nearly twice as present in the model plant Arabidopsis (Table S1), implying species or lineage-specific expansion and contraction. The coding sequence (CDS) length of *CeLEA1* genes varies from 291 to 444 bp, putatively encoding 96–147 amino acids (AA) with a small molecular weight (MW) of 10.75–15.18 kilodalton (kDa). Without any exception, all CeLEA1s possess a grand average of hydropathicity (GRAVY) of less than 0, varying from −0.780 to −1.331 (Table 1), which is in accordance with the hydrophilic feature of this family [7]. Nevertheless, analyzing the hydropathicity scale revealed the difference between CeLEA1-1/-2 and CeLEA1-3/-4/-5. Whereas hydrophobic AA are confined to the C-terminal of CeLEA1-1/-2, they were also found near the N-terminal of CeLEA1-3/-4/-5 (Figure S1). Except for Trp, the other 19 AA were found in at least one of five proteins, which are rich in Ala, Glu, Gly, Lys, and Thr. Notably, Cys and Phe are only present in CeLEA1-2 and CeLEA1-4, respectively, whereas Asn is only absent from CeLEA1-2 (Figure 1A).

To uncover their evolutionary relationships, an unrooted phylogenetic tree was constructed using the full-length protein sequences of *LEA_1* genes present in tigernut and Arabidopsis. As shown in Figure 1B, these proteins were clustered into two main groups. In Group I, two members for each species were observed, which are grouped in species, i.e., *CeLEA1-1*/*-2* and *AtLEA6*/*-18* with sequence similarities of 55.22% and 50.75% at the protein level, respectively (Table S2). On the contrary, Group II includes *AtLEA46* and three tigernut members, i.e., *CeLEA1-3*/*-4*/*-5* with 37.16%–46.27% protein similarities (Table S2). The result implies that the last common ancestor of monocots and eudicots may possess only two members, followed by species or lineage-specific expansion in tigernut and Arabidopsis. Indeed, interspecific synteny analysis revealed that *AtLEA6*/*-18*, *CeLEA1-1*/*-2*, and *CeLEA1-3*/*-4*/*-5* are located within syntenic blocks, supporting their whole-genome duplication (WGD) derivation (Table 1 and Table S1). Interestingly, except for *CeLEA1-2* that has no intron, other members in tigernut feature a single one, which is similar to that observed in Arabidopsis, where only *AtLEA18* is intronless. Notably, *CeLEA1-1* was shown to possess a phase 1 intron, in stark contrast to other genes feature a phase 0 intron (Figure 1C), implying their different origin.

Due to fast evolution, the overall pairwise sequence similarity within the family was shown to be relatively low in both tigernut and Arabidopsis, varying from 30.59% to 55.22% (Table S2). Nevertheless, all of them possess one LEA_1 domain at the N-terminal (Table 1), which is mainly represented by Motif1 identified via MEME analysis (Figure 1D). By contrast, high diversity was observed at the C-terminal, which possesses nine motifs with sequence specificity. Interestingly, Motif2 and -3, which are characterized as the C-terminal of the LEA_1 domain, are specific to Groups I and II, respectively. Nevertheless, Motif3 is absent from both CeLEA1-4 and -5 (Figure 1D), supporting their divergence.

### 2.2. Comparative Genomics Analyses Reveal Lineage-Specific Evolution of the LEA_1 Family in the Monocot Clade

To gain insights into the origin and evolution of *CeLEA1* genes, homologs were also identified from representative plant species, which include the basal angiosperm *Amborella trichopoda* and three early diverging monocots, i.e., *Acorus gramineus*, eelgrass (*Zostera marina*), and duckweed (*Spirodela polyrhiza*). In contrast to *A. trichopoda* without a recent WGD [26], it has been well established that core monocots represented by rice (*Oryza sativa*) experienced three WGDs after the split with the eudicot clade, i.e., τ, ρ, and σ [27]. Whereas the σ WGD is Poaceae specific, the ρ WGD is specific to Poales (including tigernut) and the τ WGD was estimated to occur within a window of 110–135 MYA, sometime after the split of Alismatales and commelinids but before the divergence of Asparagales and commelinids [28,29]. As shown in Table S1, a total of 120 family members were obtained. Notably, compared with a previous study [5], one more member named *OsLEA1-4* was identified in rice, whose orthologs were widely found in other Poaceae plants examined in this study (see below). Additionally, four members were identified from the transcriptome of purple nutsedge (*C. rotundus*), a species without an available genome that is close to tigernut [22]. Surprisingly, despite the presence of two members (each for Groups I and II, respectively) in both *A. trichopoda* and *A. gramineus*, no homolog was detected in eelgrass, whereas only one Group II member was identified in duckweed (Table S1), implying lineage-specific gene loss.

**Figure 2:**
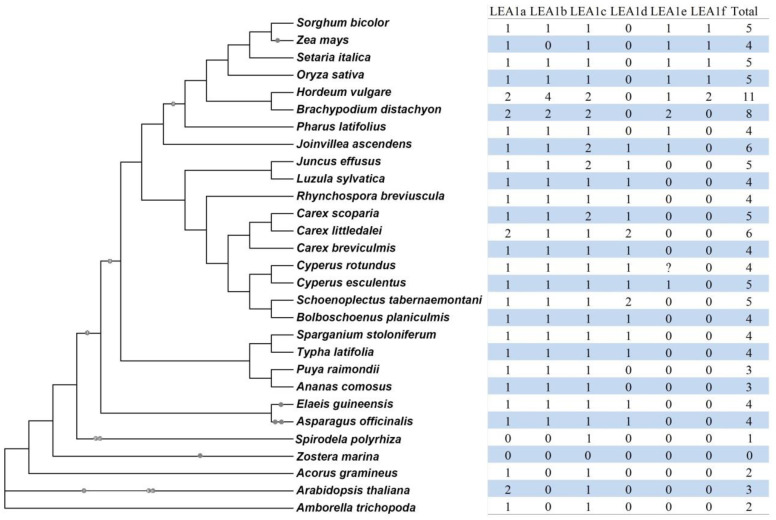
Species-specific distribution of six orthogroups in 29 representative plant species. The species tree is referred to NCBI Taxonomy (https://www.ncbi.nlm.nih.gov/taxonomy (accessed on 20 August 2023)) and recent whole-genome duplications or triplications resulting in polyploidy (CoGepedia; https://genomevolution.org/wiki/index.php/Plant_paleopolyploidy (accessed on 20 August 2023)) are marked. “?” indicates unknown. (LEA: late embryogenesis abundant.)

To infer lineage-specific evolution, orthologs between different species were identified using OrthoFinder [30], which resulted in six orthogroups (OG). Surprisingly, despite the presence of four to six members in seven Cyperaceae plants examined in this study, no clear ortholog was found for *CeLEA1-5*, in contrast to one to two that were identified in *Joinvillea ascendens* and six out of seven tested Poaceae plants, i.e., *Pharus latifolius*, *Brachypodium distachyon*, barley (*Hordeum vulgare*), rice, foxtail millet (*Setaria italica*), sorghum (*Sorghum bicolor*), and maize (*Zea mays*). Compared with Cyperaceae, one more orthogroup (i.e., LEA1f) was identified in most Poaceae plants with the exception of *P. latifolius* and *B. distachyon* (Figure 2). The presence of LEA1a, LEA1b, LEA1c, and LEA1d in garden asparagus (*Asparagus officinalis*) and oil palm (*Elaeis guineensis*) implies their early origin, most likely from the τ WGD, whereas LEA1e and LEA1f may arise from ρ and σ WGDs (see below), respectively.

**Figure 3:**
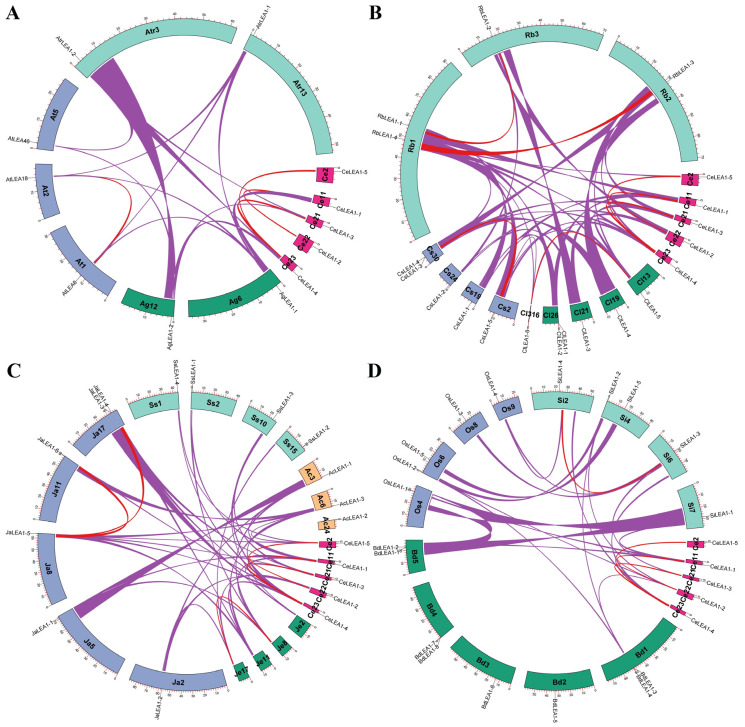
Synteny analysis within and between *C. esculentus* and representative plant species. (**A**) Synteny analysis within and between *C. esculentus*, *A. gramineus*, *A. thaliana*, and *A. trichopoda*. (**B**) Synteny analysis within and between *C. esculentus*, *C. littledalei*, *C. scoparia*, and *R. breviuscula*. (**C**) Synteny analysis within and between *C. esculentus*, *J. effusus*, *J. ascendens*, *S. stoloniferum*, and *A. comosus*. (**D**) Synteny analysis within and between *C. esculentus*, *B. distachyon*, *O. sativa*, and *S. italica*. *LEA_1* gene-encoding chromosomes/scaffolds and only syntenic blocks containing *LEA_1* genes are marked, where red and purple lines for intra- and inter-species, respectively. The scale is in Mb. (Ac: *A. comosus*; Ag: *A. gramineus*; At: *A. thaliana*; Atr: *A. trichopoda*; Bd: *B. distachyon*; Ce: *C. esculentus*; Cl: *C. littledalei*; Cs: *C. scoparia*; Ja: *J. ascendens*; Je: *J. effuses*; Mb: megabase; Os: *O. sativa*; Rb: *R. breviuscula*; Si: *S. italic*; Ss: *S. stoloniferum*).

According to interspecific synteny analysis, all *CeLEA1* genes were shown to have syntelogs in at least one out of 27 species examined in this study, which includes one in *A. trichopoda* and two in *A. gramineus*. Interestingly, despite over a long time of evolution, 1:1 and 1:2 syntenic relationships were observed between *A. trichopoda* and *A. gramineus*/Arabidopsis (Figure 3A), where *AtLEA6* and *-18* were shown to arise from the Brassicaceae-specific α WGD [10]. Nevertheless, no syntelog was identified for all five *CeLEA1* genes in Arabidopsis. Moreover, no syntenic orthologs were identified for *CeLEA1-2* and *-5* in both *A. trichopoda* and *A. gramineus* (Figure 3A), implying their late origin. Synteny analysis within representative Cyperaceae plants is shown in Figure 3B. Except for *CeLEA1-5* that only possesses a syntelog in *Rhynchospora breviuscula* (i.e., *RbLEA1-4*), other members in tigernut exhibit 1:1 and 1:2 syntenic relationships with that of *Carex littledalei*, *C. scoparia*, and *R. breviuscula*. Similar to *CeLEA1-1*/*-2* and *CeLEA1-3*/*-4*/*-5*, *RbLEA1-1*/*-2* and *RbLEA1-3*/*-4* are also located within syntenic blocks. By contrast, only *CsLEA1-3*/*-5* and *ClLEA1-5*/*-6* are still located within syntenic blocks, implying species-specific chromosome rearrangement of regions encoding *CsLEA1-1*/*-2* and *ClLEA1-1*/*-3*. An interesting result of synteny analysis within and between representative Poales plants is that *JaLEA1-3*/*-5*, *JaLEA1-5*/*-6*, *CeLEA1-3*/*JaLEA1-3*, *CeLEA1-3*/*JaLEA1-5*, *CeLEA1-4*/*JaLEA1-3*, *CeLEA1-4*/*JaLEA1-5*, *CeLEA1-4*/*JaLEA1-6*, *CeLEA1-5*/*JaLEA1-5*, *CeLEA1-5*/*SsLEA1-4*, *CeLEA1-5*/*AcLEA1-3* are located within syntenic blocks (Figure 3C), further supporting WGD-derivation of *CeLEA1-5*, most likely from the σ WGD. Notably, compared with *J. ascendens*, relatively less syntelogs were identified for *CeLEA1* genes in Poaceae plants, i.e., 5 vs. 2–3. Interestingly, though no syntenic relationship was observed within all five *OsLEA1* genes, *SiLEA1-3* and *-4* were shown to locate within syntenic blocks, providing the direct evidence that LEA1f was derived from LEA1c. Moreover, *SiLEA1-3*/*OsLEA1-3*, *SiLEA1-3*/*OsLEA1-4*, *SiLEA1-4*/*OsLEA1-4* are also located within syntenic blocks, implying species-specific chromosome rearrangement of the region encoding *OsLEA1-3* (Figure 3D). In addition to WGD, tandem duplication also played a role in the gene expansion in *C. littledalei*, *C. scoparia*, *Schoenoplectus tabernaemontani*, *Juncus effuses*, barley, and *B. distachyon*, i.e., *ClLEA1-1*/*-2*, *CsLEA1-3*/*-4*, *StLEA1-3*/*-4*, *JeLEA1-3*/*-4*, *HvLEA1-1*/*-2*, *HvLEA1-5*/*-6*, *BdLEA1-1*/*-2*, *BdLEA1-3*/*-4*, and *BdLEA1-7*/*-8* (Table S1).

Divergence of exon–intron structure was also observed. Despite the presence of one intron in both *AtrLEA1-1* and *-2*, Groups I and II members in monocots usually feature no and a single intron, respectively (Table S1). Interestingly, a phase 1 intron present in *CeLEA1-1* was also found in its orthologs of all Cyperaceae and Typhaceae species examined in this study, implying its gain sometime after the split with Joinvilleaceae but before Cyperaceae-Typhaceae divergence. On the contrary, an intron widely found in LEA1c and LEA1d is absent from Poaceae species (Table S1). Since all LEA1f members are also intronless, loss of the intron may occur sometime after the split with Joinvilleaceae but before Poaceae radiation.

Taken together, above results showed that the LEA_1 family has diverged into two phylogenetic groups before angiosperm radiation. In the monocot clade, the family was firstly expanded via the τ WGD, forming four orthogroups (i.e., LEA1a, LEA1b, LEA1c, and LEA1d), which were followed by lineage-specific expansion and contraction, especially in Poales. Whereas the ρ WGD contributed to LEA1e found in tigernut as well as Joinvilleaceae and Poaceae species, the σ WGD resulted in LEA1f, which is Poaceae specific. Gain of an intron in LEA1a is specific to Cyperaceae and Juncaceae, whereas loss of the intron in LEA1c and LEA1f is specific to Poaceae.

### 2.3. LEA_1 Genes in Tigernut Exhibit a Tuber-Predominant Expression Pattern, in Contrast to the Seed-Preferential Expression in Arabidopsis, Rice, and Maize

To provide a global view of expression evolution, the tissue-specific expression profiles of *LEA_1* genes in Arabidopsis, rice, and maize were first mined from Plant Public RNA-seq Database. As shown in Figure S2, most of them exhibit a seed/embryo-preferential expression pattern. Notably, five *OsLEA1* genes are also highly abundant in the endosperm (Figure S2), implying possible neofunctionalization.

**Figure 4:**
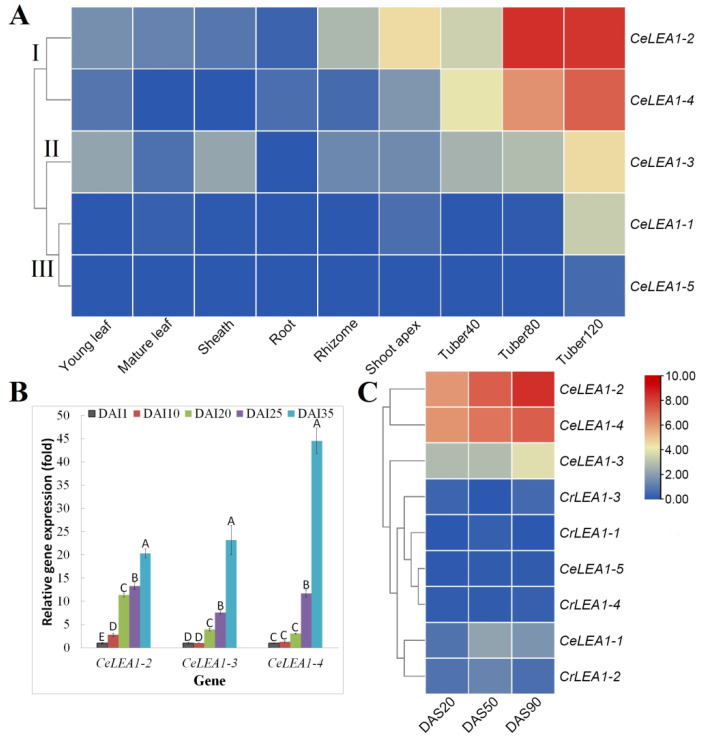
Expression profiles of *CeLEA1 and CrLEA1* genes. (**A**) Tissue-specific expression profiles of five *CeLEA1* genes. (**B**) Expression profiles of *CeLEA1-2*, *-3*, and *-4* at different stages of tuber development. (**C**) Expression profiles of *CeLEA1 and CrLEA1* genes at three representative stages of tuber development. The heatmap was generated using the R package implemented with a row-based standardization. Color scale represents FPKM normalized log_2_ transformed counts, where blue indicates low expression and red indicates high expression. Bars indicate SD (N = 3) and uppercase letters indicate a difference significance following Duncan’s one-way multiple-range post hoc ANOVA (*p* < 0.01). (Ce: *C. esculentus*; Cr: *C. rotundus*; DAI: days after tuber initiation; DAS: days after sowing; FPKM: Fragments per kilobase of exon per million fragments mapped.)

Expression profiles of *CeLEA1* genes were investigated in nine tissues/developmental stages, i.e., two stages of developmental leaf (i.e., young and mature), leaf sheath, root, rhizome, shoot apex, and three representative stages of developmental tuber, i.e., 40, 80, and 120 days after sowing (DAS), representing tuber initiation, swelling, and maturation. As shown in Figure 4A, the expression of all five *CeLEA1* genes was detected in at least one of the tissues examined, though the transcript level is highly distinct. Interestingly, most of them exhibited an apparent tuber-predominant expression pattern, where *CeLEA1-5* transcripts were only detected in tubers of 120 DAS, corresponding to no transcript present in the full-length transcriptome [24]. According to their expression profiles, five *CeLEA1* genes could be divided into three clades: Clade I includes *CeLEA1-1* and *-5* that were lowly expressed in most conditions considered in this study; Group II includes *CeLEA1-3* that was expressed in most tested conditions and moderately in tubers; Group III includes *CeLEA1-2* and *-4* that were highly abundant in tubers (Figure 4A). The results support expression and possible functional divergence of paralogs, and *CeLEA1-2* and *-4* have evolved into two dominant forms.

Moreover, during tuber development, a gradual increase in transcripts was observed for *CeLEA1-1*, *-2*, *-3*, and *-4* (Figure 4A). Notably, *CeLEA1-1* transcripts were not detected until 80 DAS, peaking at 120 DAS, which is consistent with its low abundance and late activation during tuber development. To confirm the result, qRT-PCR analysis was further conducted for *CeLEA1-2*, *-3*, and *-4* in five representative stages of tuber development, i.e., 1, 10, 20, 25, and 35 days after tuber initiation (DAI), where three middle stages represent early, middle, and late swelling, respectively [17]. As shown in Figure 4B, the trends are largely in accordance with the transcriptional profiling, and 2.84–20.34, 1.06–23.18, and 1.32–44.52 fold increase was observed for *CeLEA1-2*, *-3*, and *-4*, respectively. Notably, except for *CeLEA1-2*, no significant difference was found between two early stages, i.e., 1 and 10 DAI, whereas a significant increase was not observed for *CeLEA1-4* until 25 DAI (Figure 4B).

### 2.4. LEA_1 Genes in Tigernut Were Expressed More than Their Orthologs in Purple Nutsedge

As a close species that diverged with tigernut 0.3–12 million years ago, purple nutsedge is neither oil accumulating nor desiccation tolerant [31]. To uncover putative roles of *LEA_1* genes in desiccation tolerance of tigernut tubers, three representative stages of tuber development were profiled and compared, i.e., 20, 50, and 90 DAS. Since no ortholog was identified for *CeLEA1-5* from the purple nutsedge transcriptome assembly [22], the CDS of *CeLEA1-5* was used for read alignment instead. As expected, no mapping reads were found for this sequence, by contrast, 3 to 38 read counts were identified for *CrLEA1-1–4*, implying that the *CeLEA1-5* homolog has been lost from the purple nutsedge genome or was not expressed in tuber stages examined in this study. The total fragments per kilobase of exon per million fragments mapped (FPKM) summed up to 0.20, 3.29, 0.85, and 0.40 for *CrLEA1-1–4*, respectively, which is comparative to 0.16 for *CeLEA1-5*, but considerably lower than 8.64, 1287.07, 37.61, and 309.91 for *CeLEA1-1–4*, respectively (Figure 4C), implying species-specific activation in tigernut. Interestingly, the expression patterns of *CeLEA1* genes during tuber development are highly consistent with above transcriptional profiling and qRT-PCR analysis, though different varieties, developmental stages, and growth conditions were adopted.

## 3. Discussion

LEA proteins comprise a large superfamily that play indispensable roles in stress responses and the acquisition of desiccation tolerance of orthodox seeds [1,14,15]. Though they were first described as accumulating in late embryogenesis, *LEA* genes have been shown to express in a wide range of plant tissues, including leaves, roots, and tubers [7,10,12,32]. Moreover, whereas some of them are constitutively expressed, others were shown to be tissue specific or specifically induced by hormone and/or stress treatments [5,7,32]. Seed-preferential expression and significant roles of *LEA_1* genes have prompted us to study this special gene family in tigernut, whose underground tubers could tolerate a considerably low water content of less than 6% without affecting the sprouting capability [17,31].

### 3.1. Expansion of the LEA_1 Family in Tigernut Was Contributed by WGDs

Compared with three members reported in Arabidopsis [7], an unexpected high number of five *LEA_1* family genes were identified from the tigernut genome. Interestingly, the family numbers are comparative to four to six found in seven other Cyperaceae plants, i.e., *C. rotundus*, *C. scoparia*, *C. littledalei*, *C. breviculmis*, *R. breviuscula*, *S. tabernaemontani*, and *Bolboschoenus planiculmis*, implying lineage-specific expansion. The hypothesis was supported by interspecific synteny analysis, which revealed WGD derivation of three members, i.e., *CeLEA1-2*, *-4*, and *-5*. To uncover the evolution pattern of this family, 125 members identified from 29 species were included for comparative analysis, which represent 13 plant families, i.e., Cyperaceae (8), Juncaceae (2), Joinvilleaceae (1), Poaceae (7), Typhaceae (2), Bromeliaceae (2), Arecaceae (1), Asparagaceae (1), Zosteraceae (1), Araceae (1), Acoraceae (1), Brassicaceae (1), and Amborellaceae (1). The results showed that the *LEA_1* family has diverged into two phylogenetic groups (I and II) before angiosperm radiation, and gene expansion in tigernut was contributed by two WGD events, i.e., τ and ρ [27]. The τ WGD, which is shared by core monocots [28,29], resulted in four out of six orthogroups identified in this study, i.e., LEA1a, LEA1b, LEA1c, and LEA1d. Later in the Poales lineage, the order-specific ρ WGD further gave rise to LEA1e, which is still retained in tigernut, *J. ascendens*, and all Poaceae species examined in this study. Moreover, the Poaceae-specific σ WGD gave birth to LEA1f, which is present in rice, barley, foxtail millet, sorghum, and maize. Interestingly, despite the presence of each for two phylogenetic groups in both *A. trichopoda* and *A. gramineus*, gene loss was observed in eelgrass (0) and duckweed (1), two early diverging aquatic monocots, corresponding to the protection functions of this family in water deficit [13].

### 3.2. LEA_1 Genes in Tigernut Underwent Apparent Expression and Function Divergence

Another interesting result is that expression and function divergence was observed for *CeLEA1* genes. Consistent with previous studies [5,7], large-scale transcriptional profiling showed that *LEA_1* genes in Arabidopsis, rice, and maize exhibit a seed-preferential expression pattern, corresponding to their key roles in embryonic development [13,14,15]. Since tigernut rarely sets seeds, we are not able to analyze the expression profiles of *CeLEA1* genes during seed development. Interestingly, transcriptional profiling of 27 libraries representing six tissues (i.e., leaf, sheath, root, rhizome, shoot apex, and tuber) revealed that *CeLEA1* genes have evolved to predominantly express in oil-rich tubers, supporting their neofunctionalization. Unlike most tubers (e.g., purple nutsedge) and tuberous roots (e.g., *Manihot esculenta*) [17,31,33], tigernut experiences the seed-like desiccation during maturation, which is accompanied by significant accumulation of lipid droplets and related structural proteins such as oleosins [22,23,31]. According to a previous study, the full period of tuber development in Hainan province (China) spans approximately 35 d, and the moisture content maintains a relatively high level (~85%) until 25 DAI with a significant decrease to approximately 75%, followed by a further drop to approximately 48% at 35 DAI [17]. Tubers at early stages are white in color (including 20 DAI), turning brown at 25 DAI, and becoming hard and dark brown at 35 DAI [17,22]. These results suggest that tuber maturing may start at 25 DAI, spanning approximately 10 d. To uncover the putative role of *CeLEA1* genes during this process, their expression profiles were monitored in tubers of 1, 10, 20, 25, and 35 DAI. As expected, *CeLEA1* transcripts are usually low at 1 and 10 DAI, followed by rapid accumulation at three late stages, negatively correlating with the trend of the moisture content [17]. Despite the expression of all five *CeLEA1* genes in tubers at 35 DAI, their transcripts are distinct and *CeLEA1-2* and *-4* have evolved into two dominant members, implying expression divergence of paralogs. Moreover, *CeLEA1* transcripts in tubers were shown to be considerably more than that of their orthologs in purple nutsedge, another Cyperaceae plant with desiccation-sensitive tubers that is close to tigernut [20,22,31]. These results imply species-specific activation and key roles of *CeLEA1* genes especially *CeLEA1-2* and *-4* in the acquisition of desiccation tolerance of tigernut tubers as observed in orthodox seeds [1]. Interestingly, dehydration-specific induction of *LEA_1* genes in seeds was proven to be dependent on ABA INSENSITIVE 3 (ABI3) [12,14]. However, an *ABI3* homolog identified from the tigernut genome was barely expressed in tuber transcriptomes available in public databases. Thereby, a tissue-specific regulatory network may exist in tigernut tubers, which is more likely contributed by certain tuber-specific *cis*-acting elements and related transcription factors.

## 4. Conclusions

This study presents a first genome-wide analysis of the *LEA_1* family in tigernut, a Cyperaceae plant producing desiccation-tolerant tubers. Identification and comparison of 125 members from 29 plant species support early divergence of the *LEA_1* family into two phylogenetic groups before angiosperm radiation, and gene expansion in tigernut was contributed by two WGDs occurring after the split with the eudicot clade. These two phylogenetic groups have evolved to form at least six orthogroups in the momocot clade, five of which are present in tigernut and the remaining one is Poaceae specific, supporting lineage-specific evolution. Tuber-predominant expression of *CeLEA1* genes and seed desiccation-like accumulation of their transcripts (especially *CeLEA1-2* and *-4*) during tuber maturation imply their roles in the desiccation resistance of tigernut tubers just like that observed in orthodox seeds. These findings not only improve our understanding of lineage-specific evolution of the *LEA_1* family, but also provide valuable information for further functional analysis and genetic improvement in tigernut.

## 5. Materials and Methods

### 5.1. Datasets and Identification of LEA_1 Family Genes

Genome sequences of representative plant species were downloaded from Genome Warehouse (https://ngdc.cncb.ac.cn/gwh/, accessed on 20 August 2023), TAIR11 (https://www.arabidopsis.org/, accessed on 20 August 2023), Phytozome v13 (https://phytozome.jgi.doe.gov/pz/portal.html, accessed on 20 August 2023), and NCBI (https://www.ncbi.nlm.nih.gov/, accessed on 20 August 2023): *A. trichopoda* (v2.1), Arabidopsis (Araport11), *A. gramineus* (v1), eelgrass (v3.1), duckweed (v2), garden asparagus (v1.1), oil palm (v3), *Ananas comosus* (v3), *Puya raimondii* (v1), *Sparganium stoloniferum* (v1), *Typha latifolia* (v1), rice (v7.0), barley (Morex V3), *B. distachyon* (v3.2), foxtail millet (v2.2), sorghum (v5.1), maize (RefGen_V4), tigernut (v1), *C. littledalei* (v1), *C. breviculmis* (v1), *C. scoparia* (v1), *S. tabernaemontani* (v1), and *B. planiculmis* (v1). Transcriptome data of tigernut were accessed from NCBI (https://www.ncbi.nlm.nih.gov/, accessed on 20 August 2023), whereas for purple nutsedge, the de novo assembled transcriptome described before [25] was adopted. To identify *LEA_1* family genes, HMMER (v3.3.2, http://hmmer.janelia.org/, accessed on 20 August 2023) searches were performed using the Pfam profile PF03760 (v35.0, https://pfam.xfam.org/, accessed on 20 August 2023). Gene models of candidates were manually revised with mRNAs when available, whereas gene structures were displayed using GSDS 2.0 [34]. Presence of the conserved LEA_1 domain in deduced proteins was confirmed using Pfam Search (http://pfam.xfam.org/, accessed on 20 August 2023), and biochemical parameters were calculated using ProtParam (http://web.expasy.org/protparam/, accessed on 20 August 2023). Expression data of Arabidopsis, rice, and maize were accessed from Plant Public RNA-seq Database (https://plantrnadb.com/, accessed on 20 August 2023).

### 5.2. Phylogenetic and Conserved Motif Analyses

Multiple sequence alignments were carried out using MUSCLE implemented in MEGA (v6) [35], and phylogenetic tree construction was performed using MEGA6 with the maximum likelihood method and bootstrap of 1000 replicates. Conserved motifs were identified using MEME (v5.4.1, https://meme-suite.org/tools/meme, accessed on 20 August 2023) with the parameters as follows: any number of repetitions; maximum number of motifs, 10; and, the optimum width of each motif, between 5 and 50 residues.

### 5.3. Synteny Analysis and Definition of Orthogroups

Synteny analysis was conducted as previously described [36], and different modes of gene duplication were identified using the DupGen_finder pipeline [37]. Orthologous genes were clustered using Orthofinder (v2.3.8) [30].

### 5.4. Plant Materials

In this experiment, a tigernut variety Reyan3 was used, and growing conditions were as previously described [18,21]. Tubers with three biological replicates were collected at 1, 10, 20, 25, and 35 DAI, which represent tuber initiation, three stages of swelling (early, middle, and late), and maturation. Once collected, all samples were frozen with liquid nitrogen and stored at −80 °C for further uses.

### 5.5. Gene Expression Analysis Based on RNA-Seq

Global expression profiles of *CeLEA1* genes were analyzed using the Illumina RNA-seq dataset (PRJNA703731, 150 bp paired-end reads), which includes nine tissues/development stages with three biological replicates, i.e., young leaf, mature leaf, sheath of mature leaf, root, rhizome, stem apex, and three stages of developmental tuber that were collected at 40, 80, and 120 DAS. Quality control of raw RNA-seq reads and subsequent read mapping were conducted as previously described [21], and relative gene expression level was presented as FPKM [38].

### 5.6. Gene Expression Analysis Based on qRT-PCR

Total RNA extraction, the integrity and concentration detection, and synthesis of the first-strand cDNA were carried out as previously described [18]. Primers used for qRT-PCR analysis are shown in Table S3, where *CeTIP41* and *CeUCE2* described before [24] were employed as two reference genes. PCR reaction in triplicate for each biological sample was conducted on a Real-time Thermal Cycler Type 5100 (Thermal Fisher Scientific Oy, Waltham, MA, USA) as described before [39]. Relative gene abundance was estimated with the 2^−ΔΔCt^ method and statistical analysis was performed using the Data Processing System software v20, where differences among means of three replicates were tested following Duncan’s one-way multiple-range post hoc ANOVA.

## Figures and Tables

**Table 1:** *LEA_1* family genes identified in *C. esculentus*. (AA: amino acid; Ce: *C. esculentus*; GRAVY: grand average of hydropathicity; kDa: kilodalton; LEA: late embryogenesis abundant; MW: molecular weight; pI: isoelectric point; Scf: scaffold; WGD: whole-genome duplication.)

Gene Name	Locus ID	Position	AA	MW (kDa)	pI	GRAVY	LEA_1 Location	Duplicate	Mode	Group
*CeLEA1-1*	CESC_14864	Scf11:2802864..2803416(−)	112	12.13	9.40	−1.137	1..70	-	-	I
*CeLEA1-2*	CESC_19809	Scf22:1436544..1436936(+)	130	13.73	9.66	−0.780	1..70	*CeLEA1-1*	WGD	I
*CeLEA1-3*	CESC_02592	Scf21:1359881..1360694(+)	147	15.18	6.59	−0.784	9..78	-	-	II
*CeLEA1-4*	CESC_10473	Scf23:2545043..2545918(+)	133	13.67	9.79	−0.934	1..70	*CeLEA1-3*	WGD	II
*CeLEA1-5*	CESC_14205	Scf2:1221799..1222572(−)	96	10.75	5.30	−1.331	1..64	*CeLEA1-4*	WGD	II

## Data Availability

Transcriptome data used in this study are under the NCBI accession number of PRJNA703731.

## Supplementary Materials

The following supporting information can be downloaded at: https://www.mdpi.com/article/10.3390/plants13202933/s1, Figure S1: Kyte–Doolittle hydrophobicity plots of CeLEA1 proteins using ProtScale. (Ce: *C. esculentus*; LEA: late embryogenesis abundant.) Figure S2: Global expression profiles of *AtLEA1*, *OsLEA1*, and *ZmLEA1* genes. (At: *A. thaliana*; Ce: *C. esculentus*; LEA: late embryogenesis abundant; Os: *O. sativa*; Zm: *Z. mays.*) Table S1: *LEA_1* family genes identified in representative plant species. (AA: amino acid; Ac: *A. comosus*; Ag: *A. gramineus*; Ao: *Asparagus officinalis*; At: *A. thaliana*; Atr: *A. trichopoda*; Bd: *B. distachyon*; Bp: *B. planiculmis*; Cb: *C. breviculmis*; Ce: *C. esculentus*; Chr: chromosome; Cl: *C. littledalei*; Cr: *C. rotundus*; Cs: *C. scoparia*; Eg: *Elaeis guineensis*; Hv: *H. vulgare*; Ja: *J. ascendens*; Je: *J. effusus*; LEA: late embryogenesis abundant; Ls: *L. sylvatica*; Pl: *P. latifolius*; Pr: *Puya raimondii*; Rb: *R. breviuscula*; Scf: scaffold; Sb: *S. bicolor*; Si: *S. italica*; Sp: *Spirodela polyrhiza*; Ss: *S. stoloniferum*; St: *S. tabernaemontani*; Tl: *T. latifolia*; WGD: whole-genome duplication; Zm: *Zea mays.*) Table S2: Percent similarity within and between CeLEA1s and AtLEA1s. (At: *A. thaliana*; Ce: *C. esculentus*; LEA: late embryogenesis abundant). Table S3: Primers used in this study. (Ce: *C. esculentus*; LEA: late embryogenesis abundant.)
