# Supplementary material for: Insights into Genes Encoding LEA_1 Domain-Containing Proteins in Cyperus esculentus, a Desiccation-Tolerant Tuber Plant
Source: Plants (Basel). 2024 Oct 19;13(20):2933. doi: 10.3390/plants13202933 (PMC11511247; doi:10.3390/plants13202933)
Supplement: Supplementary file 1 [file plants-13-02933-s001.zip › Figures S1-S2.pdf]

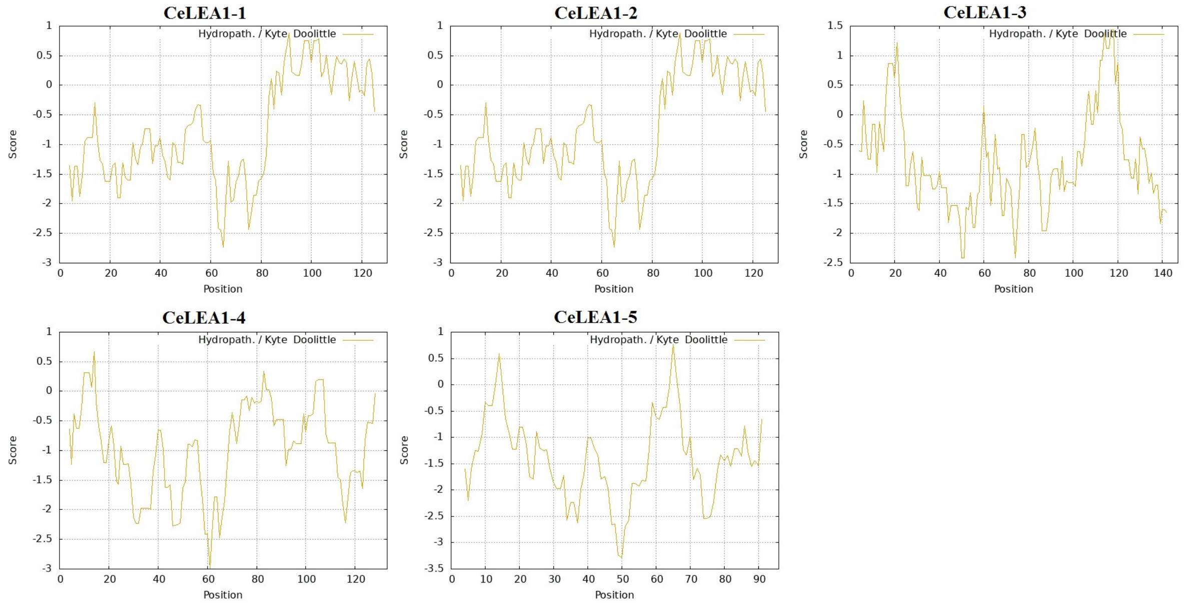

Figure S1: Kyte–Doolittle hydrophobicity plots of CeLEA1 proteins using ProtScale. (Ce: *C. esculentus*; LEA: late embryogenesis abundant.)

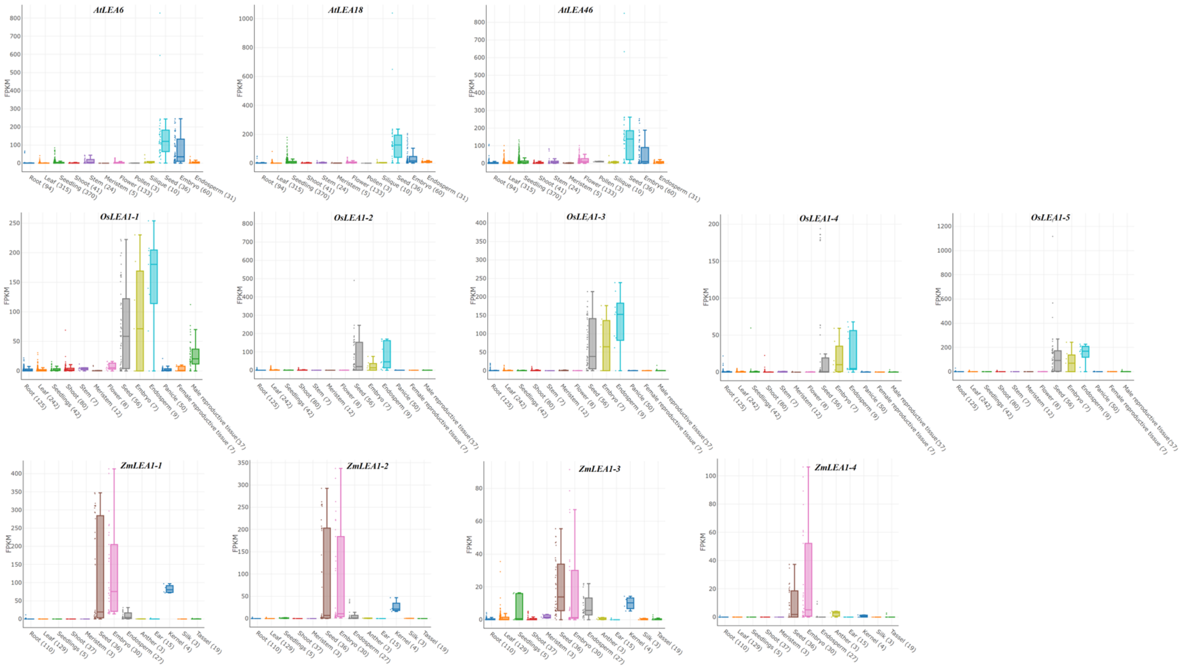

Figure S2: Global expression profiles of *AtLEA1*, *OsLEA1*, and *ZmLEA1* genes. (At: *A. thaliana*; Ce: *C. esculentus*; LEA: late embryogenesis abundant; Os: *O. sativa*; Zm: *Z. mays*.)
